# Identification of a pyroptosis-related lncRNA risk model for predicting prognosis and immune response in colon adenocarcinoma

**DOI:** 10.1186/s12957-022-02572-8

**Published:** 2022-04-12

**Authors:** Yuying Tan, Liqing Lu, Xujun Liang, Yongheng Chen

**Affiliations:** 1 Department of Oncology, NHC Key Laboratory of Cancer Proteomics, XiangYa Hospital, Central South University, Changsha, 410008 China; 2 National Clinical Research Center for Geriatric Disorders, XiangYa Hospital, Central South University, Changsha, 410008 China

**Keywords:** Colon adenocarcinoma, ceRNA network, Pyroptosis, Tumor immune microenvironment, Immunotherapy

## Abstract

**Background:**

Colon adenocarcinoma (COAD) is one of the most common malignant tumors and is diagnosed at an advanced stage with a poor prognosis worldwide. Pyroptosis is involved in the initiation and progression of tumors. This research focused on constructing a pyroptosis-related ceRNA network to generate a reliable risk model for risk prediction and immune infiltration analysis of COAD.

**Methods:**

Transcriptome data, miRNA-sequencing data, and clinical information were downloaded from the TCGA database. First, differentially expressed mRNAs (DEmRNAs), miRNAs (DEmiRNAs), and lncRNAs (DElncRNAs) were identified to construct a pyroptosis-related ceRNA network. Second, a pyroptosis-related lncRNA risk model was developed applying univariate Cox regression analysis and least absolute shrinkage and selection operator method (LASSO) regression analysis. Kyoto Encyclopedia of Genes and Genomes (KEGG) and Gene Ontology (GO) enrichment analyses were utilized to functionally annotate RNAs contained in the ceRNA network. In addition, Kaplan-Meier analysis, receiver operating characteristic (ROC) curves, univariate and multivariate Cox regression, and nomogram were applied to validate this risk model. Finally, the relationship of this risk model with immune cells and immune checkpoint blockade (ICB)-related genes was analyzed.

**Results:**

A total of 5373 DEmRNAs, 1159 DElncRNAs, and 355 DEmiRNAs were identified. A pyroptosis-related ceRNA regulatory network containing 132 lncRNAs, 7 miRNAs, and 5 mRNAs was constructed, and a ceRNA-based pyroptosis-related risk model including 11 lncRNAs was built. The tumor tissues were classified into high- and low-risk groups according to the median risk score. Kaplan-Meier analysis showed that the high-risk group had a shorter survival time; ROC analysis, independent prognostic analysis, and nomogram further indicated the risk model was a significant independent prognostic factor what had an excellent ability to predict patients’ risk. Moreover, immune infiltration analysis indicated that the risk model was related to immune infiltration cells (i.e., B cell naïve, T cell follicular helper, macrophage M1) and ICB-related genes (i.e., *PD-1*, *CTLA4*, *HAVCR2*).

**Conclusions:**

This pyroptosis-related lncRNA risk model possessed good prognostic value, and the ability to predict the outcome of ICB immunotherapy in COAD.

**Supplementary Information:**

The online version contains supplementary material available at 10.1186/s12957-022-02572-8.

## Introduction

Colorectal cancer (CRC) is the most common cancer diagnosed in the world [1]. The incidence and mortality rates of CRC are in the top three of all cancers based on the American Cancer Society 2021 report [1]. Colon adenocarcinoma (COAD) is the most common histological subtype of CRC. With the advancements in the diagnosis and treatment of COAD in recent years, its incidence and mortality remain at 10.2% and 9.2%, respectively [2]. Therefore, the improvement of early diagnosis and treatment modalities for COAD patients is an urgent clinical need.

Pyroptosis is a gasdermin-mediated inflammatory programmed cell death characterized by cell swelling, pore formation, and the release of intracellular contents, such as IL-1β and IL-18 [3]. Pyroptosis is typically triggered by canonical pathways and non-canonical pathways [4, 5]. In the past several years, an increasing number of studies have depicted that pyroptosis is involved in the progression of cancer. The primary therapeutic strategy of cancer is to induce cell death, and some researchers are trying to find novel targeted therapies for COAD by activating pyroptosis pathways [6].

The competitive endogenous RNA (ceRNA) hypothesis, including non-coding RNAs and mRNAs, is considered as a novel regulatory network, which reveals a novel mechanism of interaction between RNAs. These ceRNA molecules can compete to bind the same miRNA through microRNA response elements (MRE) to affect the gene expression [7]. Long non-coding RNA (lncRNA), more than 200 nucleotides in length, is defined as a non-coding RNA and has been found to be involved in diverse key biological processes, including cell proliferation and differentiation, genetic regulation of gene expression, and regulation of microRNAs (miRNAs) [8]. Studies have shown that lncRNAs can disrupt the balance of the ceRNA network, thereby promoting cancer progression [9, 10]. For example, Ma et al. depicted that the lncRNA RP1-85F18.6 was upregulated in CRC and played major roles in tumorigenesis and repressed the pyroptosis of CRC cells [11]. In addition, several studies have described that lncRNAs promote tumorigenesis by changing the immune microenvironment in cancers [12, 13]. To date, the pyroptosis-related ceRNA networks have not been elucidated in COAD.

In this study, transcriptome and miRNA sequencing data between COAD tumor tissues and normal tissues were retrieved from The Cancer Genome Atlas (TCGA) database (https://portal.gdc.cancer.gov/). The pyroptosis-related ceRNA network was constructed using integrated analysis. A pyroptosis-related prognostic signature was extracted from the ceRNA network. Then, we investigated the role of this pyroptosis-related lncRNA prognostic signature in immune microenvironment and immune checkpoint inhibitor treatment.

## Materials and methods

### Data acquisition

RNA-seq data of COAD was derived from the TCGA-COAD dataset including 398 tumor samples and 39 normal samples. The miRNA data was downloaded from the TCGA including 380 tumor samples and 8 normal samples. Meanwhile, relevant clinical data of 385 COAD patients was also obtained from TCGA, including age, gender, survival status, stage, T, N, and M classification (Table 1).

**Table 1:** Characteristics of the COAD patients obtained from the TCGA database

Characteristic	TCGA_COAD (***n***=385)
**Age (years)**	
≤65	159 (41.30%)
>65	226 (58.70)
**Gender**	
Male	205 (53.25%)
Female	180 (46.75%)
**Survival status**	
Alive	314 (81.56%)
Dead	71 (18.44%)
**Stage**	
Stage I	66 (17.14%)
Stage II	151 (39.22%)
Stage IIII	103 (26.75%)
Stage IV	54 (14.03%)
Unknown	11 (2.86%)
**T classification**	
This	1 (0.26%)
T1	9 (2.34%)
T2	68 (17.66%)
T3	263 (68.31%)
T4	44 (11.43%)
**N classification**	
N0	231 (60.00%)
N1	88 (22.86%)
N2	66 (17.14%)
**M classification**	
M0	286 (74.28%)
M1	54 (14.03%)
Unknown	45 (11.69%)

### Identification of differentially expressed genes (DEGs)

The R package “edgeR” was applied to identify the DEGs of lncRNAs (DElncRNAs), mRNAs (DEmRNAs), and miRNAs (DEmiRNAs) with a false discovery rate (FDR) adjusted *P*<0.05 and |log2FC|>1.0 as the cutoff criterion [14]. Then, the volcano maps and heatmaps were plotted employing “ggplot2” and “pheatmap” R packages [15].

### Construction of a pyroptosis-related ceRNA network

The miRcode database was applied to predict the interaction pairs between DElncRNAs and DEmiRNAs [16]. The DEmiRNA-targeted mRNAs (miTGs) were obtained from TargetScan, miRTarBase, and miRDB databases [17, 18]. In addition, the pyroptosis-related DEmRNAs were retrieved by the intersection of miTGs, DEmRNAs, and 155 pyroptosis-related genes (PRGs) from GeneCards (https://www.genecards.org/, Supplement Table S1). Finally, a pyroptosis-related ceRNA network was visualized using Cytoscape v3.8.2.

### Identification and validation of a ceRNA-based pyroptosis-related lncRNA risk model

A total of 379 COAD patients with survival data were included. One hundred thirty-two pyroptosis-related lncRNAs (PRlncRNAs) in the above ceRNA network were analyzed by univariate Cox regression analysis to filter PRlncRNAs associated with survival. To avoid overfitting, PRlncRNAs were screened via least absolute shrinkage and selection operator (LASSO) regression analysis (R package “glmnet”, *P*<0.05) [19, 20]. Then, a PRlncRNA risk model was constructed and the risk score of each sample was calculated based on the formula below: risk score = ∑_i_Xi × Yi (X: coefficients, Y: lncRNA expression level) [21]. And tumor tissues were separated into high- and low-risk groups according to the median risk score. Next, lncRNAs in the PRlncRNA risk model with associated DEmiRNAs and DEmRNAs were used to construct a prognostic ceRNA network. To assess the putative biological role of RNAs in the ceRNA network, Gene Ontology (GO) and Kyoto Encyclopedia of Genes and Genomes (KEGG) functional enrichment analyses were conducted (R packages “clusterProfiler”, “org.Hs.eg.db,” and “enrichplot”) [22].

### Validation of the risk model

Kaplan-Meier analysis was used to compare the overall survival (OS) time between the two risk groups using R packages “survival” and “survminer” [23]. The 1-, 3-, and 5-year receiver operating characteristic (ROC) curves were acquired by utilizing the “timeROC”, “survival,” and “survminer” R packages [24]. The area under the receiver operating curve (AUC) was used to indicate forecast performance. Independent prognostic analysis showed the relationship of risk score and clinical traits through the “survival”, “survminer,” and “forestplot” R packages [23].

### Establishment of the nomogram

A nomogram was established to quantitatively calculate patient survival. Then, calibration curves were employed to confirm the predictive effect of nomogram (R package “rms”).

### Immune cell infiltration and immune checkpoint blockade (ICB) analysis

To further investigate the difference between immune cell infiltration in the two risk groups, the relationship between the immune cell proportion and risk score was estimated by the CIBERSORT algorithm (*P*<0.05). The Spearman correlation analysis was employed to estimate the association of the immune cells and risk score.

The expression level of ICB-related genes was closely involved with the outcome of immunotherapy. Therefore, the Spearman correlation between the 11 ICB-related genes and risk score was analyzed [25].

### Statistical analysis

All statistical analyses were performed using Perl v5.32.1 and R 4.1.0 software. The ceRNA network was constructed by Cytoscape v3.8.2. The Wilcoxon test was used to compare the proportion of immune cells between the two groups. DEmRNAs, DElncRNAs, and DEmiRNAs were gained with the thresholds FDR <0.05 and |log2 fold change|>1. Statistical tests were two-tailed (*P*<0.05).

## Results

### DEmRNAs, DElncRNAs, and DEmiRNAs

**Fig. 1:**
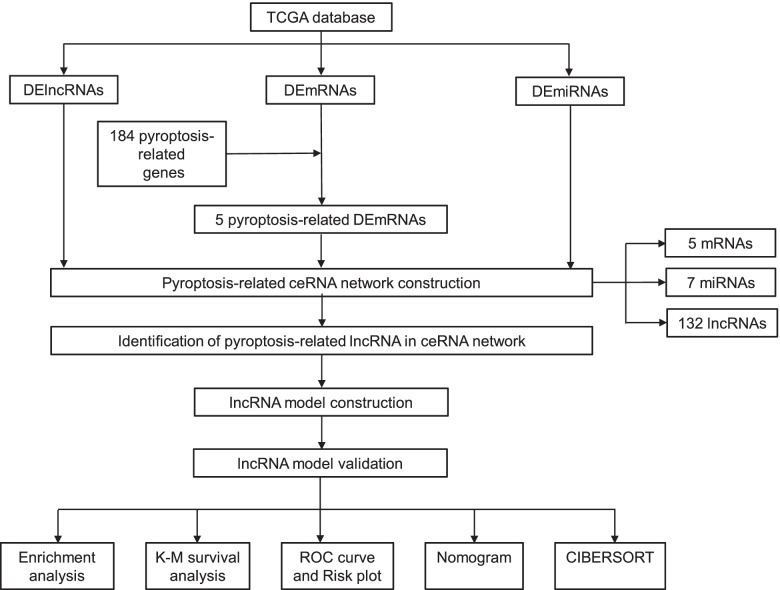
The analysis process of the study. TCGA, The Cancer Genome Atlas; DElncRNAs, differentially expressed lncRNAs; DEmRNAs, differentially expressed mRNAs, DEmiRNAs, differentially expressed miRNAs; ceRNA, competitive endogenous RNA; CIBERSORT, cell-type identification by estimating relative subsets of RNA transcripts

**Fig. 2:**
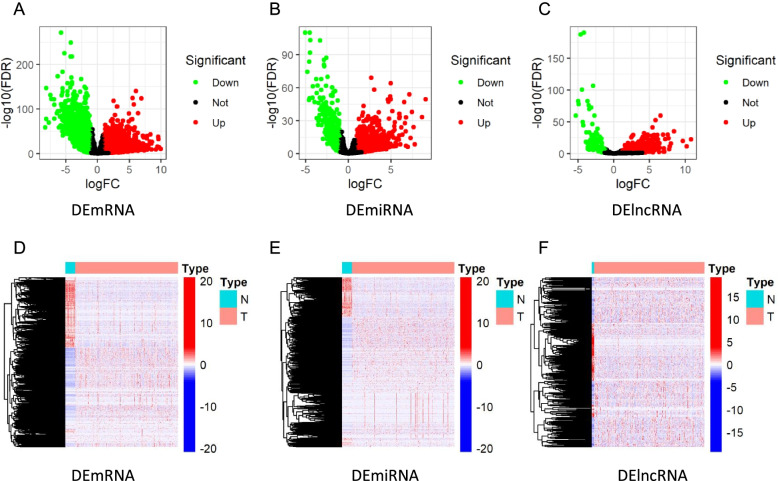
Differentially expressed genes. Volcano plots of **A** DEmRNAs, **B** DEmiRNAs, and **C** DElncRNAs. Heatmaps of **D** DEmRNAs, **E** DEmiRNAs, and **F** DElncRNAs

The flow diagram of the current study is shown in Fig. 1. We identified 5373 DEmRNAs (2886 upregulated and 2487 downregulated), 355 DEmiRNAs (217 upregulated and 138 downregulated), and 1159 DElncRNAs (819 upregulated and 340 downregulated) for further analysis in the TCGA database of COAD with FDR<0.05 and |log2FC|>1.0 criteria. The volcano maps and heatmaps showed the DEmRNA, DEmiRNA, and DElncRNA expression of COAD, respectively (Fig. 2A–F).

### Construction of a pyroptosis-related ceRNA network in COAD

To construct the pyroptosis-related ceRNA network that facilitates understanding of the links between DEmRNAs, DElncRNAs, and DEmiRNAs, several databases were used.

**Fig. 3:**
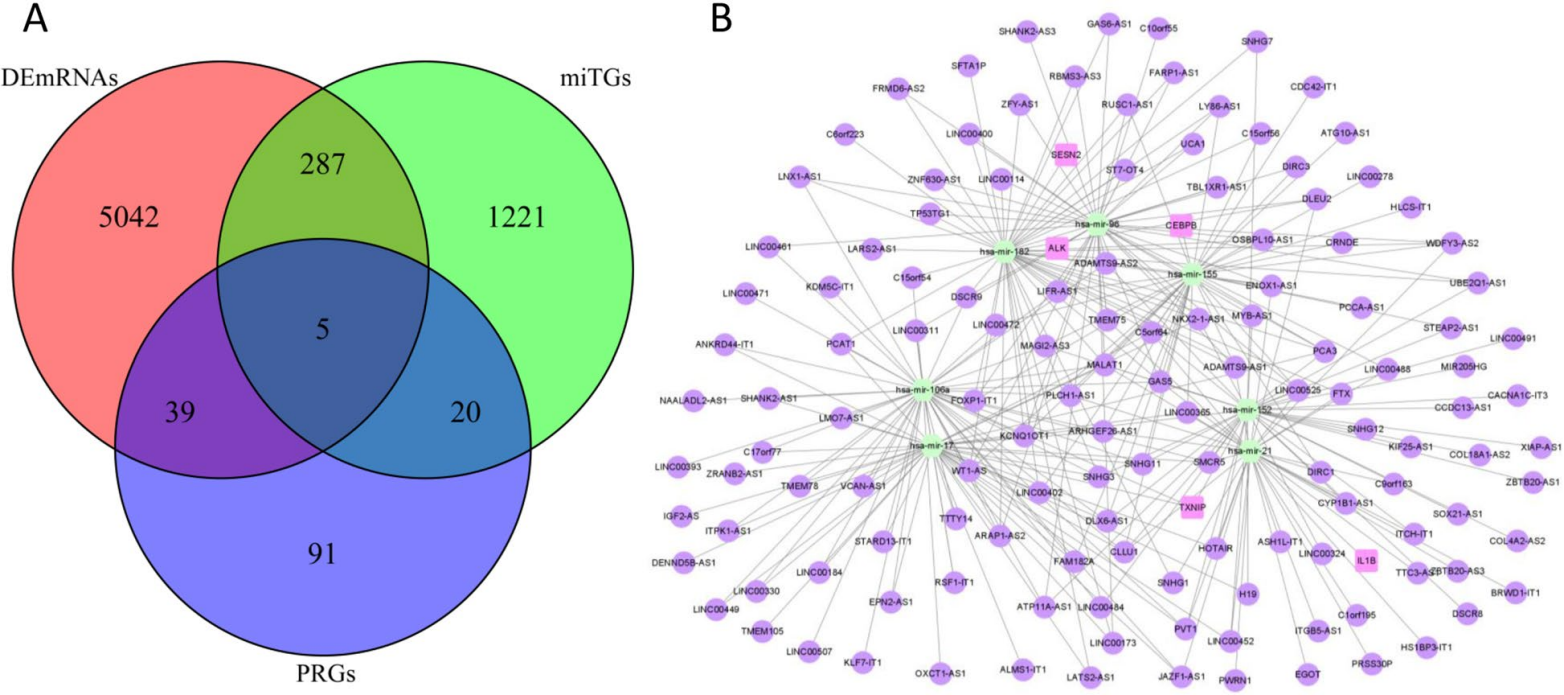
The pyroptosis-related ceRNA network. **A** The intersection of DEmRNAs, miTGs, and PRGs. **B** A pyroptosis-related ceRNA network. Lavender circles indicate pyroptosis-related DEmRNAs, green circles indicate DEmiRNAs, and purple circles indicate DElncRNAs

Based on the miRcode database, the lncRNA-miRNA interactions including 218 DElncRNAs and 38 DEmiRNAs were identified. The miRNA-mRNA interactions including 38 DEmiRNAs and 1533 miTGs were acquired based on the TargetScan, miRTarBase, and miRDB databases. Then, we screened 5 pyroptosis-related genes by taking the intersection of 1533 miTGs, 5373 DEmRNAs, and 155 pyroptosis-related genes (Fig. 3A). Finally, 5 DEmRNAs, 7 DEmiRNAs, and 132 DElncRNAs were gained, then we constructed a pyroptosis-related ceRNA network of COAD (Fig. 3B, Supplement Table S2).

### Construction of a PRlncRNA prognostic risk model

**Fig. 4:**
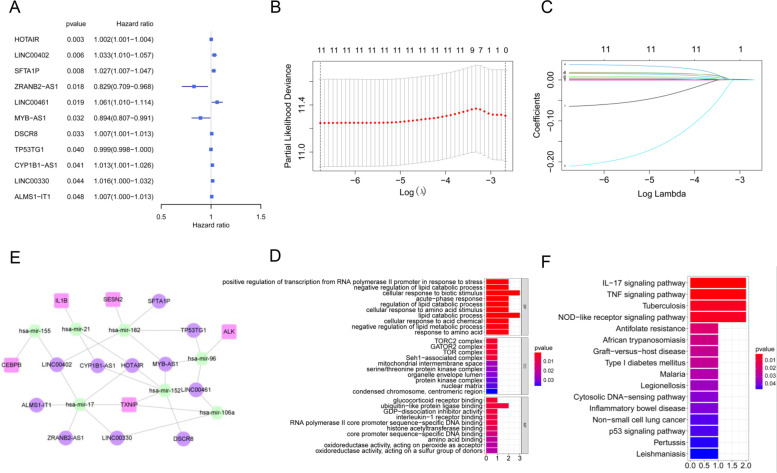
The PRlncRNA risk model. **A** The univariate Cox regression of selected lncRNAs (criterion: *P* value < 0.05). **B**, **C** The LASSO Cox regression analysis of lncRNAs with *P*<0.05. **D** A ceRNA network of 11 lncRNAs with 7 miRNAs and 5 mRNAs based on the prognostic PRlncRNA risk model. **E**, **F** GO and KEGG enrichment analyses of genes included in the above ceRNA network

To build the pyroptosis-related lncRNA model for forecasting the overall survival of COAD patients, 132 DElncRNAs were employed to construct the prognostic model. Univariate Cox regression analysis showed that 11 lncRNAs were associated with overall survival (*P*<0.05, Supplement Table S3). Among them, 8 lncRNAs (*HOTAIR*, *LINC00402*, *SFTA1P*, *LINC00461*, *DSCR8*, *CYP1B1-AS1*, *LINC00330*, *ALMS1-IT1*) were risk genes with HR>1, and the other 3 lncRNAs (*ZRANB2-AS1*, *MYB-AS1*, *TP53TG1*) were protective genes with HR<1 (Fig. 4A, Table 2). Subsequently, LASSO regression analysis was performed, and 11 lncRNAs highly correlated with overall survival were identified, which were used to construct a pyroptosis-related lncRNA risk model (Fig. 4B, C). The risk score was computed using the following formula: risk score = (0.0013*HOTAIR exp) + (0.0174*LINC00402 exp) + (0.0186*SFTA1P exp) + (0.0373*LINC00461 exp) + (−0.2108*ZRANB2-AS1 exp) + (−0.0012* *TP53TG1* exp) + (−0.0647* *MYB-AS1* exp) + (0.0032* *DSCR8* exp) + (0.0084* *LINC00330* exp) + (0.0156* *CYP1B1-AS1* exp) + (0.0053*ALMS1-IT1 exp). According to the previous ceRNA network, 7 miRNA (hsa-mir-155, hsa-mir-21, hsa-mir-182, hsa-mir-96, hsa-mir-152, hsa-mir-17, hsa-mir-106a) and 5 mRNAs (CEBPB, IL1B, SESN2, ALK, TXNIP) were obtained, which were related to the above 11 lncRNAs. Then, we re-established a ceRNA network based on 11 lncRNAs, 7 miRNAs, and 5 mRNAs (Fig. 4D).

**Table 2:** The lncRNAs identified by univariate Cox regression with *P* value<0.05

id	HR	HR.95L	HR.95H	*P* value
HOTAIR	1.002262	1.000763	1.003763	0.003088
LINC00402	1.033168	1.009627	1.057258	0.005525
SFTA1P	1.026793	1.006943	1.047034	0.00794
ZRANB2-AS1	0.828482	0.709043	0.96804	0.017841
LINC00461	1.060789	1.00986	1.114287	0.018733
MYB-AS1	0.894277	0.807239	0.9907	0.032451
DSCR8	1.006876	1.000555	1.013238	0.032954
TP53TG1	0.998724	0.997509	0.999939	0.039597
CYP1B1-AS1	1.013262	1.00052	1.026166	0.041305
LINC00330	1.015966	1.000451	1.031721	0.043648
ALMS1-IT1	1.006703	1.000045	1.013406	0.048477

*HR* Hazard ratio

### Functional enrichment analysis of pyroptosis-related ceRNA genes

To explore potential functions of these 11 lncRNAs, 7 miRNAs, and 5 mRNAs in biological processes, GO and KEGG functional enrichment analyses were performed with *P* value < 0.05 as the threshold. For GO analysis, these genes were primarily enriched in the cellular response to biotic stimulus and lipid catabolic process in biological processes (BPs); TORC2 complex, GATOR2 complex, and TOR complex in cellular components (CCs); and ubiquitin-like protein ligase binding in molecular function (MF) (Fig. 4E). KEGG pathway analysis suggested that these genes were markedly concentrated in the IL-17 signaling pathway, TNF signaling pathway, tuberculosis, and NOD-like receptor signaling pathway (Fig. 4F).

### Validation of the prognostic PRlncRNA risk model

**Fig. 5:**
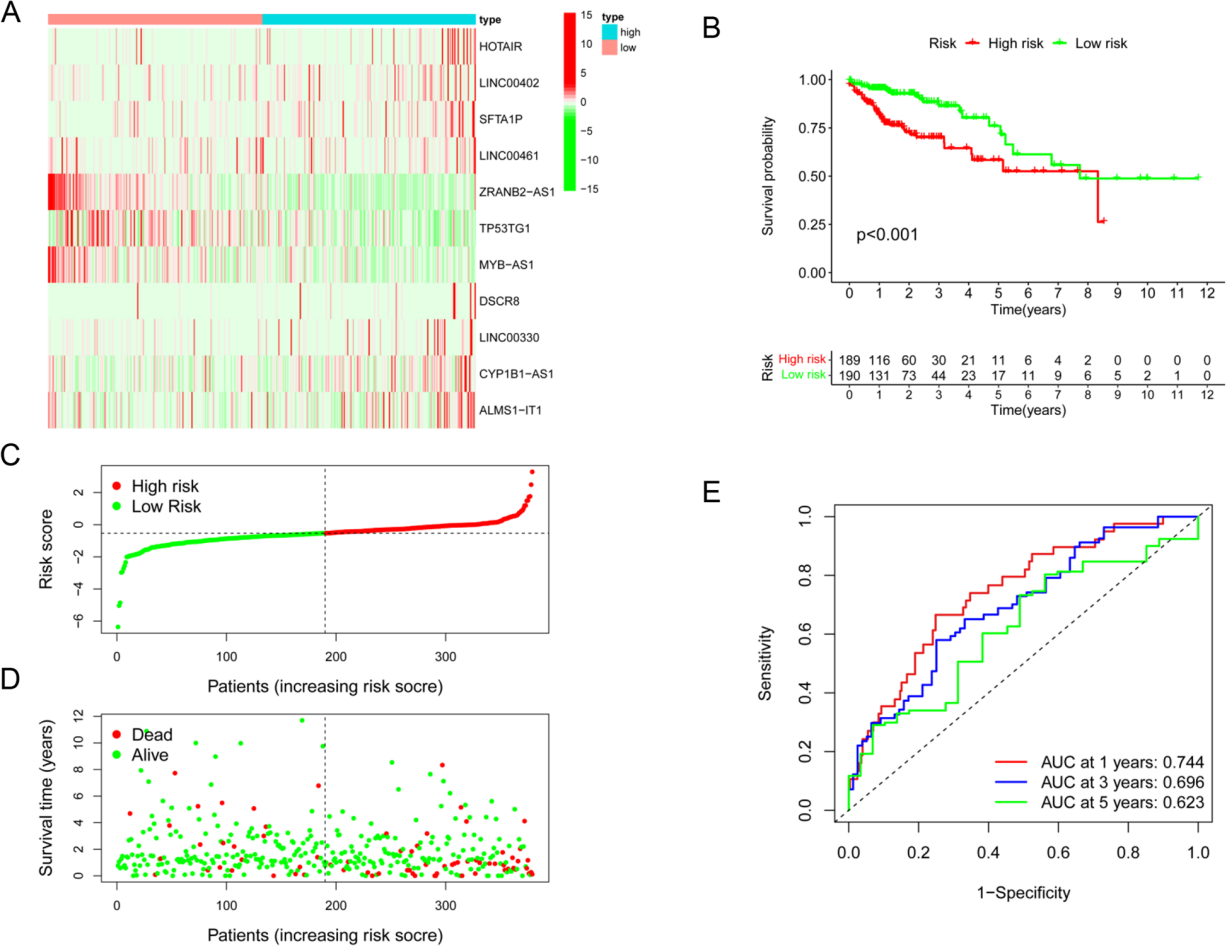
Validation of the PRlncRNA risk model. **A** Heatmap of 11 prognostic lncRNAs between the two risk groups where red presents high expression level and green presents low expression level. **B** K-M analysis. **C** Distribution of the risk score. **D** The survival status of COAD patients. **E** ROC curves of 1-, 3-, and 5-year OS time

In the prognostic PRlncRNA risk model, 8 lncRNAs (*HOTAIR*, *LINC00402*, *SFTA1P*, *LINC00461*, *DSCR8*, *CYP1B1-AS1*, *LINC00330*, *ALMS1-IT1*) were upregulated, while the other 3 lncRNAs (*ZRANB2-AS1*, *MYB-AS1*, *TP53TG1*) were downregulated in tumor tissues (Fig. 5A). To determine the potential prognostic capability of the PRlncRNA risk model in predicting COAD patient overall survival, COAD patients were categorized into high- or low-risk groups in terms of the median risk value. Kaplan-Meier analysis showed that patients in the high-risk group had shorter survival times (Fig. 5B). Likewise, patients were separated into high-risk and low-risk groups based on the median risk score (Fig. 5C). As the risk score increased, the patient’s survival time decreased gradually (Fig. 5D). ROC analysis was used to evaluate the predictive power of this prognostic model, which indicated that the PRlncRNA risk model was able to excellently predict the 1-year (0.744), 3-year (0.696), and 5-year (0.623) survival of COAD patients, respectively (Fig. 5E).

### Independent prognostic analysis of the prognostic PRlncRNA risk model

**Fig. 6:**
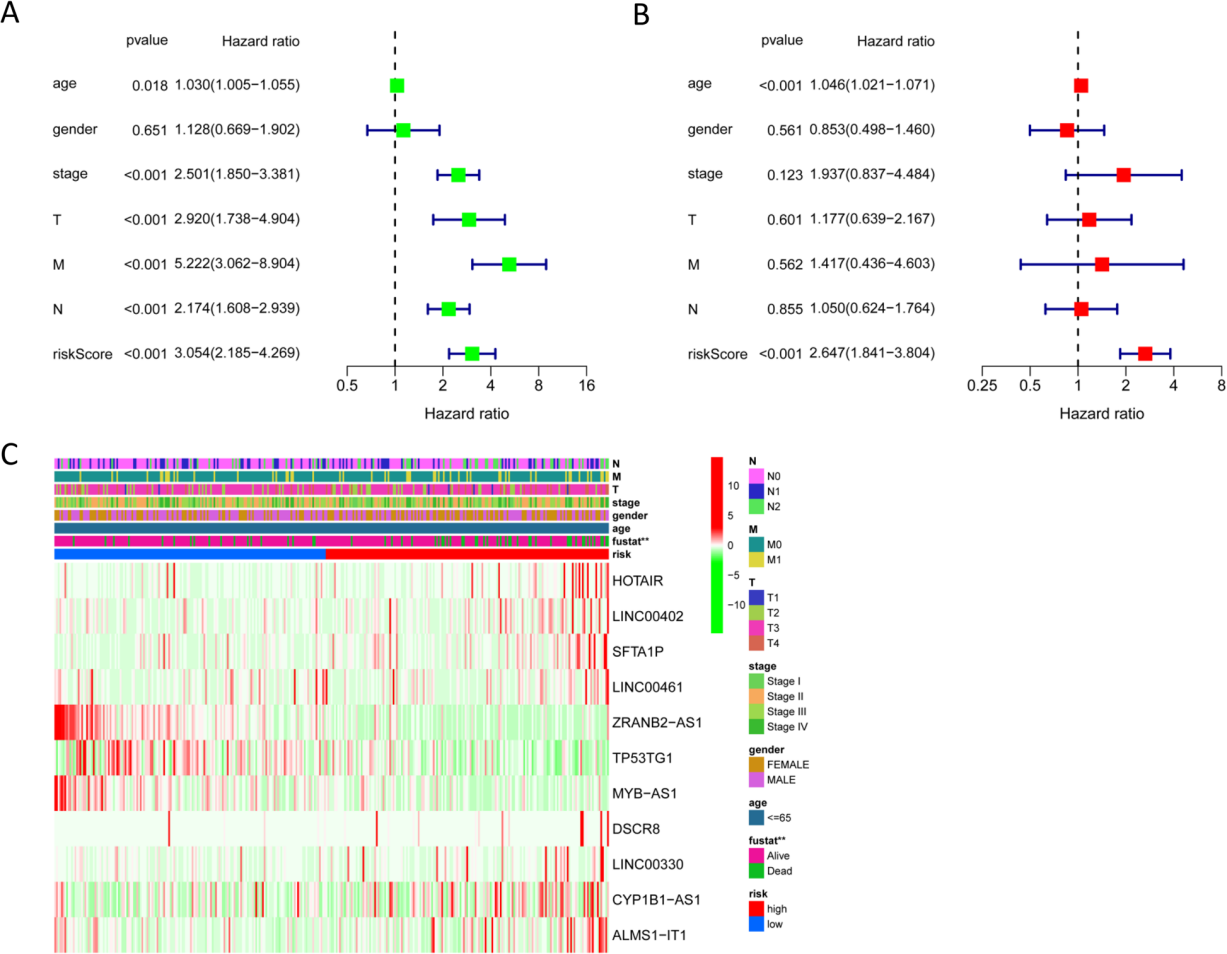
Independent prognostic analysis. **A** Univariate Cox analysis. **B** Multivariate Cox analysis. **C** Heatmap of the correlation of the risk model and clinical characteristics (*P* value** < 0.01, *P* value* < 0.05)

To explore whether the prognostic PRlncRNA risk model can independently predict the prognosis of COAD, univariate and multivariate Cox regression analyses were employed. The univariate Cox regression analysis showed that age (*P* = 0.018), clinical stage (*P*<0.01), T stage (*P*<0.001), risk score (*P*<0.001), N stage (*P*<0.001), and M stage (*P*<0.028) predicted dismal OS (Fig. 6A). And the results of multivariate Cox regression analysis confirmed the independence of the prognostic PRlncRNA risk model for predicting COAD prognosis (Fig. 6B). Moreover, the heatmap of clinical characteristics implied that the survival status of patients was differentially distributed between the low- and high-risk subgroups (*P*<0.05, Fig. 6C).

### Construction of a predictive nomogram

**Fig. 7:**
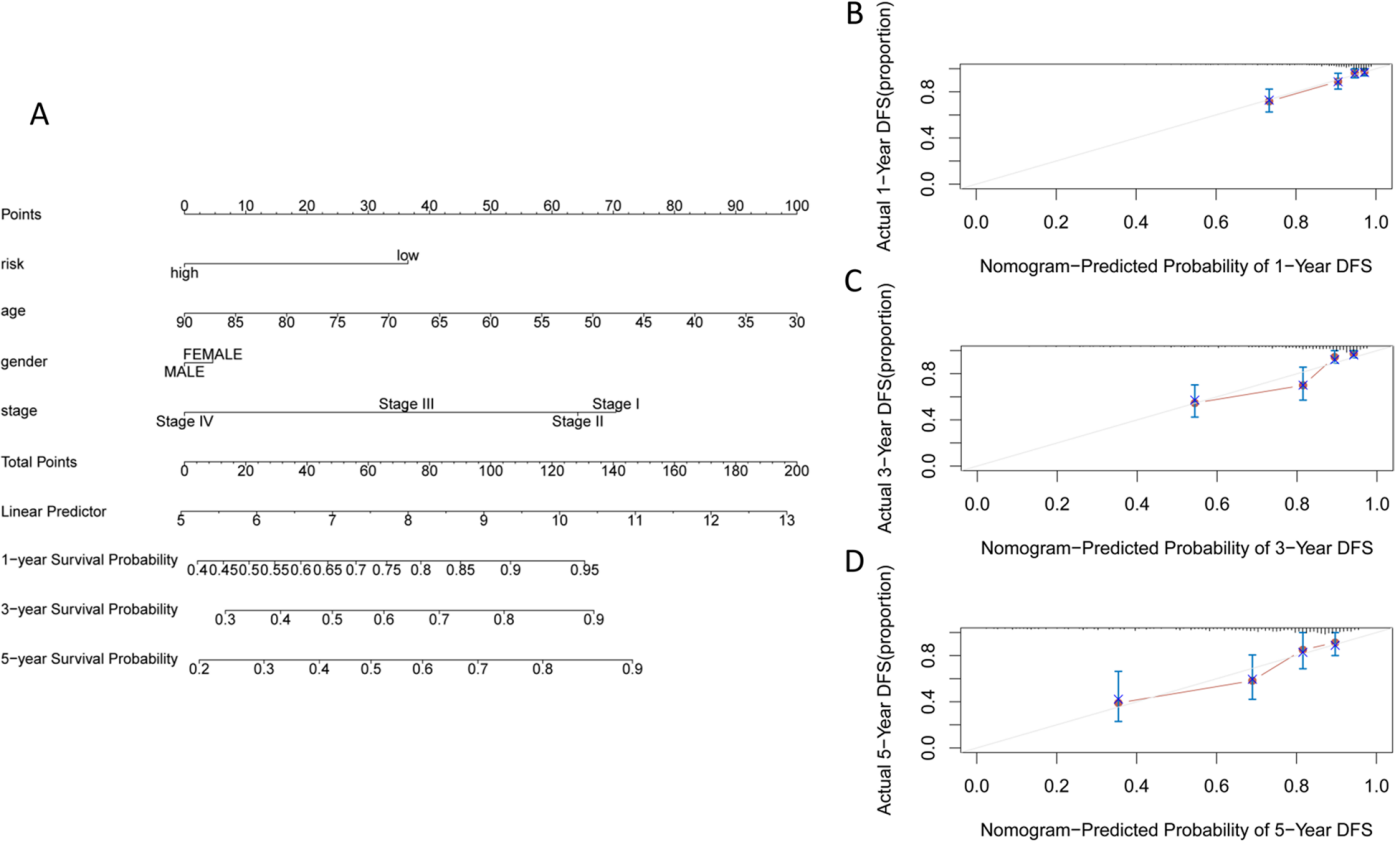
Validation of predictive ability of the risk model. **A** The nomogram contained risk values, age, and stage to predict survival. **B** One-year nomogram calibration curve. **C** Three-year nomogram calibration curve. **D** Five-year nomogram calibration curve

To further assess whether this prognostic PRlncRNA risk model had optimal predictive capabilities, we collected clinical characteristics, including age, gender, and stage, as candidate predictive biomolecular indicators to construct a nomogram (Fig. 7A). The calibration curves of 1 year, 3 years, and 5 years suggested that the nomogram was close to the actual morality and had good predictive power (Fig. 7B–D). The above findings showed a promising capacity of the PRlncRNA risk model for patient prognosis and survival prediction.

### Immunoinfiltration analysis

**Fig. 8:**
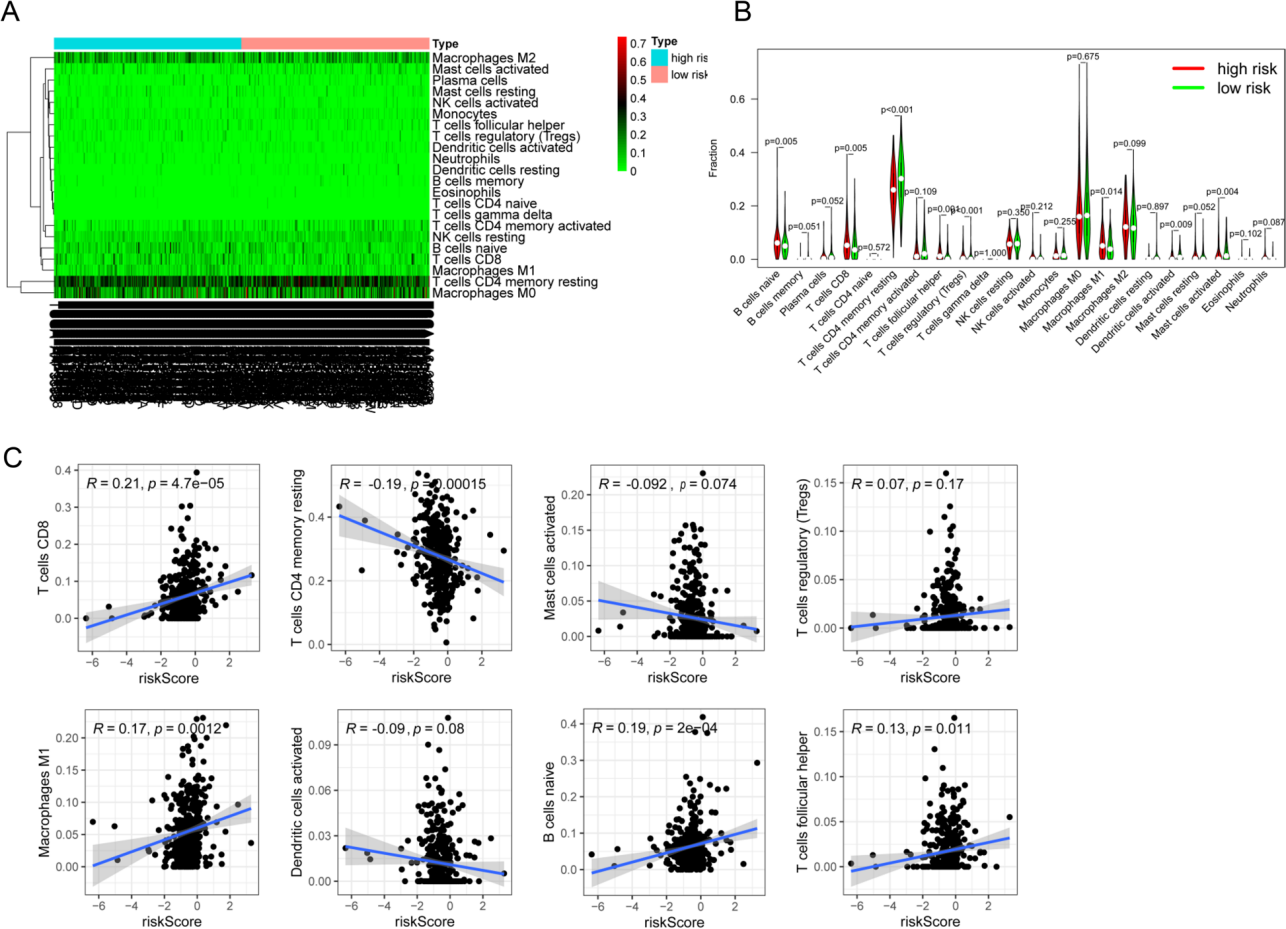
Immune cell infiltration analysis. **A** Heatmap of the composition of the immune cells was calculated based on the CIBERSORT algorithm. **B** The proportion of immune cells in the two risk groups (*P*<0.05). **C** Correlation analysis between the risk score and 8 immune cells (*P*<0.05)

Studies have shown that the pyroptosis of tumor cells can effectively regulate the tumor immune microenvironment (TIME) and activate a strong T cell-mediated anti-tumor immune response [26]. To further explore the relationship between this PRlncRNA risk model and TIME, the CIBERSOR algorithm was utilized to compare the proportions of 22 immune infiltrating cells between the high-risk and low-risk groups. B cell naïve, T cell CD8, T cell follicular helper, T cell regulatory (Tregs), and macrophage M1 were higher expressed while T cell CD4 memory resting, dendritic cells activated, and mast cells activated were lower expressed in the high-risk group by using Wilcoxon rank-sum test (Fig. 8A, B). These results provided strong evidence that our prognostic risk model was significantly associated with immune cells infiltration in COAD. Additionally, we performed correlation analysis between the prognostic risk model with the above eight significantly different immune infiltrating cells, and the results showed that the risk score was positively related to the immune infiltration of B cells naïve (*R*=0.19, *P*=2e−04), macrophages M1 (*R*=0.17, *P*=0.0012), T cell CD8 (*R*=0.21, *P*=4.7e−05), and T cells follicular helper (*R*=0.13, *P*=0.011), while negatively associated with T cell CD4 memory resting infiltration (*R*=−0.19, *P*=0.00015) (Fig. 8C), indicating that this risk model might be a key part in the assessment of responsiveness to ICB immunotherapy in COAD.

### Relationship of the prognostic PRlncRNA risk model and immune checkpoint blockades (ICBs)

**Fig. 9:**
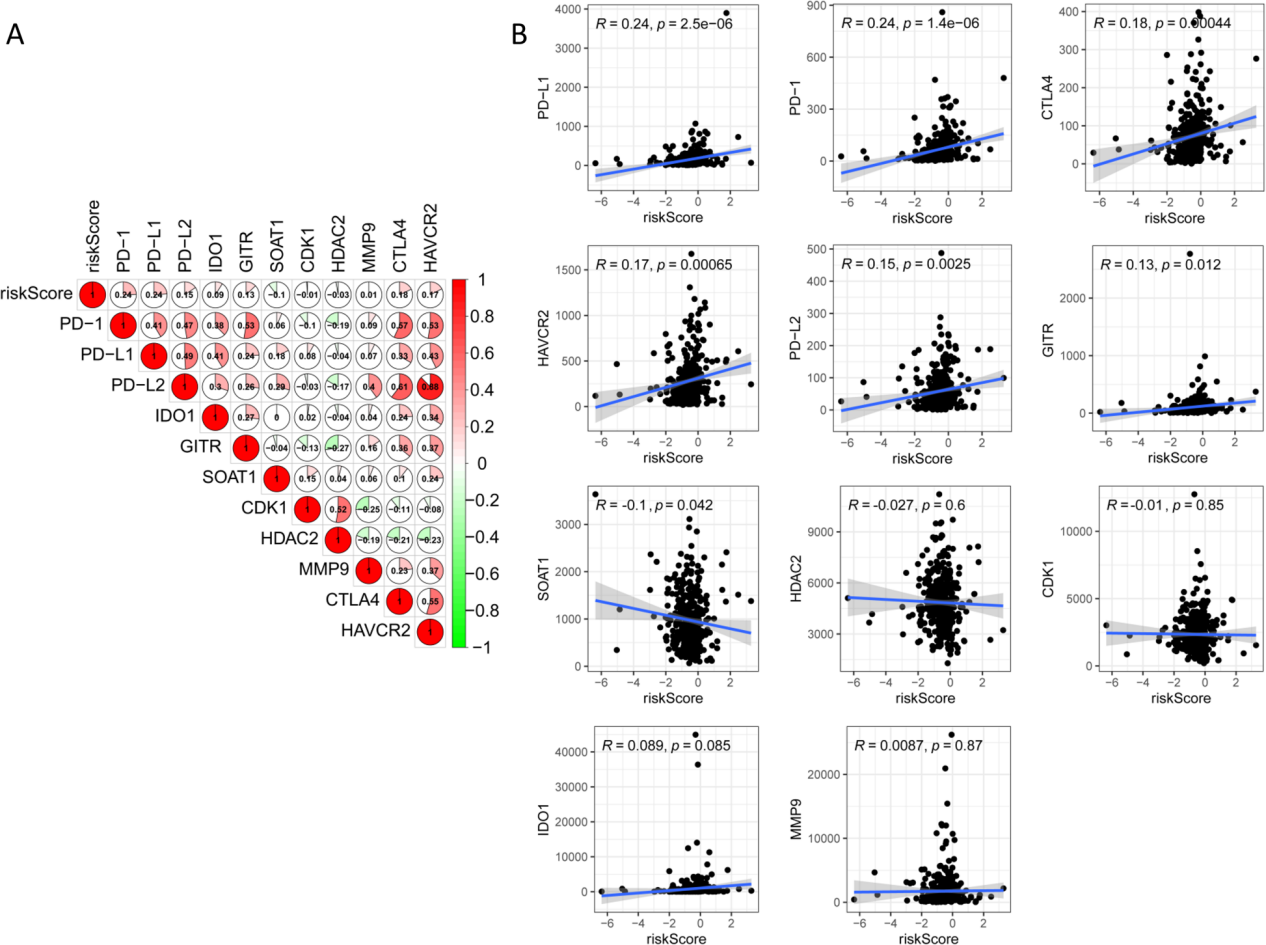
The connection between the prognostic risk score and ICB-related genes. **A** Heatmap of the correlation of the risk score and 11 ICB-related genes. **C** The connection between the risk score and 11 ICB-related genes, respectively (*P*<0.05)

ICB-related immunotherapy has become a promising modality for patients with COAD [27]. To investigate the response of COAD samples to immunotherapy, we examined the association of ICB-related genes (i.e., *PD-1*, *CTLA4*, *HAVCR2*) and risk score (Fig. 9A). The results showed that the risk score was significantly positively correlated with *PD-1* (*R*=0.245, *P*=1.44e−06), *PD-L1* (*R*=0.24, *P*=2.5e−06), *PD-L2* (*R*=0.15, *P*=0.0025), *GITR* (*R*=0.13, *P*=0.012), *HAVCR2* (*R*=0.17, *P*=0.00065), and *CTLA4* (*R*=0.18, *P*=0.00044), but negatively associated with *SOAT1* (*R*=−0.1, *P*=0.042), indicating that the risk score model was useful in assessing patients’ response to immunotherapy (Fig. 9B).

## Discussion

Colon adenocarcinoma (COAD) is the most common malignancy with increased mortality worldwide [28]. Despite significant improvements in surgery, radiotherapy, chemotherapy, and immunotherapy, the rates of 5-year survival remain low [29]. Therefore, it is crucial to identify potential biomarkers for the diagnosis and treatment of COAD. Pyroptosis was regarded as a new type of programmed cell death that played a dual function in the development of cancer [3]. In recent years, pyroptosis has become a hot topic in the field of oncology research, and an increasing number of researches have emphasized the key effects of pyroptosis in tumorigenesis and TIME [26, 30, 31]. ceRNA represents a new regulation mode of gene expression, which is more sophisticated and complex than the miRNA regulatory network. There is growing evidence that the ceRNA network is involved in the progression of COAD [32, 33]. Nevertheless, the underlying function of the ceRNA-based pyroptosis-related risk model in prognostic prediction and tumor immunity of COAD has not yet been elucidated, and our study was designed to clarify this role.

Our study constructed a pyroptosis-related ceRNA network and PRlncRNA risk model. Moreover, we also explored the prognostic predictive ability of the risk model and its association with immune cell infiltration and assessed the reactivity of COAD patients to ICB therapy.

In current research, a pyroptosis-related ceRNA regulatory network including 5 mRNAs, 7 miRNAs, and 11 lncRNAs was first constructed to investigate the potential molecular mechanism of the ceRNA network. Furthermore, GO and KEGG enrichment analyses showed that these genes were mostly enriched in the IL-17 signaling pathway and TNF signaling pathway. These results suggested that this pyroptosis-related ceRNA network can be a new tool to predict clinical results of COAD. However, these discoveries need to be verified in additional studies. Moreover, 11 pyroptosis-related lncRNAs were incorporated into developing a PRlncRNA risk model. Then, Kaplan–Meier curve, time-dependent ROC curves, Cox regression analysis, and nomogram showed that this risk model possessed excellent prediction ability and became an independent predictor of COAD prognosis.

A growing body of evidence has demonstrated that lncRNAs play an essential role in regulating immune cell infiltration [34, 35], and some researches have also reported that pyroptosis of tumor cells regulates tumor-suppressed immune cells [26, 31]. Furthermore, we investigated the fundamental effects of the risk score in the regulation of the TIME. Consistent with the previous reports, our research also showed that the risk score was negatively correlated with resting immune cell proportions, but positively associated with immunosuppressive cells, indicating that patients with low-risk scores were immunologically resting, while those with high-risk scores represent an immunosuppressive tumor microenvironment.

With the development of immune checkpoint inhibitors, ICB immunotherapy has generated promising therapeutic results in COAD [36, 37]. To date, immunotherapy has become the fifth pillar in the foundation of COAD therapeutics. Unfortunately, the majority of COAD patients do not respond to ICB treatment [38]. Pyroptosis can alter the immune microenvironment and remodel immune cells to enhance the efficiency of tumor immunotherapies [39]. Previous researches have demonstrated that pyroptosis induction plus PD-1 improves the anti-tumor activity [26, 40]. Thus, a novel PRlncRNA risk model was developed to investigate the correlation of pyroptosis and ICB-related genes and to predict COAD patients’ responses to ICB immunotherapy. The current study has shown that the PRlncRNA risk model was strongly related to ICB-related genes (i.e., PD-1, PD-L1), which implied that the PRlncRNA risk model could be utilized to evaluate the response to ICB treatment of COAD patients. Meanwhile, further validation of the PRlncRNA risk model as a useful predictor of immune checkpoint therapy in COAD is needed in the future.

Our research has some limitations. All analyses were employed by using the TCGA-COAD cohort, which would be better to validate with other database cohorts. In addition, in vivo and in vitro experiments should be conducted to further verify our results. However, the novelty of our study is that for the first time, the molecular mechanism of COAD was investigated from the perspective of the pyroptosis-related ceRNA network. Additionally, 11 pyroptosis-related lncRNA prognostic biomarkers were screened based on the ceRNA network. Moreover, the PRlncRNA risk model possessed a high predictive ability for survival in COAD patients. This may provide a new idea for the study of COAD.

## Conclusions

To sum up, we performed comprehensive and systematic bioinformatics analysis and constructed a PRlncRNA risk model for COAD patients, which could be used as a potent tool in predicting the prognosis of COAD patients. In addition, the risk model was related to TIME- and ICB-related genes. The pyroptosis-related ceRNA network might be a promising therapeutic target in COAD.

## Supplementary Information

**Additional file 1: Table S1.** 155 pyroptosis-related genes acquired from the GeneCard.

**Additional file 2: Table S2.** 5 mRNAs, 7 miRNAs and 132 lncRNAs in the PRlncRNA ceRNA network.

**Additional file 3: Table S3.** The univariate Cox regression of 132 lncRNAs included in the PRlncRNA ceRNA risk model.

## Data Availability

This study analyzed publicly available datasets which were obtained from the following sources: mRNA expression profile, miRNA expression profile, lncRNA expression profile, and clinical information of COAD were acquired from TCGA-COAD (https://portal.gdc.cancer.gov/).

## Abbreviations

DEGs: Differentially expressed genes
FC: Fold change
FDR: False discovery rate
GO: Gene Ontology
KEGG: Kyoto Encyclopedia of Genes and Genomes
TCGA: The Cancer Genome Atlas
ROC: Receiver operating characteristic
ICB: Immune checkpoint blockade
LASSO: Least absolute shrinkage and selection operator method
ceRNA: Competitive endogenous RNA
MRE: MicroRNA response elements
PRlncRNAs: Pyroptosis-related lncRNAs

## References

[1] Siegel RL, Miller KD, Fuchs HE, Jemal A (2021). Cancer Statistics, 2021. CA Cancer J Clin.

[2] Liu Y, Li C, Dong L, Chen X, Fan R. Identification and verification of three key genes associated with survival and prognosis of COAD patients via integrated bioinformatics analysis. Biosci Rep. 2020;40(9):BSR20200141. 10.1042/BSR20200141 PMC7522359 32936304

[3] Shi J, Zhao Y, Wang K, Shi X, Wang Y, Huang H, Zhuang Y, Cai T, Wang F, Shao F (2015). Cleavage of GSDMD by inflammatory caspases determines pyroptotic cell death. Nature.

[4] Wu D, Wang S, Yu G, Chen X (2021). Cell death mediated by the pyroptosis pathway with the aid of nanotechnology: prospects for cancer therapy. Angew Chem Int Ed Engl.

[5] Hsu SK, Li CY, Lin IL, Syue WJ, Chen YF, Cheng KC, Teng YN, Lin YH, Yen CH, Chiu CC (2021). Inflammation-related pyroptosis, a novel programmed cell death pathway, and its crosstalk with immune therapy in cancer treatment. Theranostics.

[6] Yu P, Zhang X, Liu N, Tang L, Peng C, Chen X (2021). Pyroptosis: mechanisms and diseases. Signal Transduct Target Ther.

[7] Salmena L, Poliseno L, Tay Y, Kats L, Pandolfi PP (2011). A ceRNA hypothesis: the Rosetta Stone of a hidden RNA language?. Cell.

[8] Li X, Wu Z, Fu X, Han W (2013). Long noncoding RNAs: insights from biological features and functions to diseases. Med Res Rev.

[9] Peng WX, Koirala P, Mo YY (2017). LncRNA-mediated regulation of cell signaling in cancer. Oncogene.

[10] Taniue K, Akimitsu N (2021). The functions and unique features of LncRNAs in cancer development and tumorigenesis. Int J Mol Sci.

[11] Ma Y, Chen Y, Lin C, Hu G (2018). Biological functions and clinical significance of the newly identified long non-coding RNA RP1-85F18.6 in colorectal cancer. Oncol Rep.

[12] Atianand MK, Caffrey DR, Fitzgerald KA (2017). Immunobiology of long noncoding RNAs. Annu Rev Immunol.

[13] Chen YG, Satpathy AT, Chang HY (2017). Gene regulation in the immune system by long noncoding RNAs. Nat Immunol.

[14] Robinson MD, McCarthy DJ, Smyth GK (2010). edgeR: a Bioconductor package for differential expression analysis of digital gene expression data. Bioinformatics.

[15] Wu X, Sui Z, Zhang H, Wang Y, Yu Z (2020). Integrated analysis of lncRNA-mediated ceRNA network in lung adenocarcinoma. Front Oncol.

[16] Jeggari A, Marks DS, Larsson E (2012). miRcode: a map of putative microRNA target sites in the long non-coding transcriptome. Bioinformatics.

[17] Hsu SD, Lin FM, Wu WY, Liang C, Huang WC, Chan WL, Tsai WT, Chen GZ, Lee CJ, Chiu CM (2011). miRTarBase: a database curates experimentally validated microRNA-target interactions. Nucleic Acids Res.

[18] Wong N, Wang X (2015). miRDB: an online resource for microRNA target prediction and functional annotations. Nucleic Acids Res.

[19] Gupta S, Lee REC, Faeder JR (2020). Parallel tempering with Lasso for model reduction in systems biology. PLoS Comput Biol.

[20] Zhang M, Zhu K, Pu H, Wang Z, Zhao H, Zhang J, Wang Y (2019). An immune-related signature predicts survival in patients with lung adenocarcinoma. Front Oncol.

[21] Li X, Meng Y (2019). Survival analysis of immune-related lncRNA in low-grade glioma. BMC Cancer.

[22] Du X, Zhang Y (2020). Integrated analysis of immunity- and Ferroptosis-related biomarker signatures to improve the prognosis prediction of hepatocellular carcinoma. Front Genet.

[23] Wei C, Liang Q, Li X, Li H, Liu Y, Huang X, Chen X, Guo Y, Li J (2019). Bioinformatics profiling utilized a nine immune-related long noncoding RNA signature as a prognostic target for pancreatic cancer. J Cell Biochem.

[24] Li M, Liang M, Lan T, Wu X, Xie W, Wang T, Chen Z, Shen S, Peng B (2020). Four immune-related long non-coding rnas for prognosis prediction in patients with hepatocellular carcinoma. Front Mol Biosci.

[25] Chen R, Chen Y, Huang W, Zhao Y, Luo W, Lin J, Wang Z, Yang J (2021). Comprehensive analysis of an immune-related ceRNA network in identifying a novel lncRNA signature as a prognostic biomarker for hepatocellular carcinoma. Aging (Albany NY).

[26] Wang Q, Wang Y, Ding J, Wang C, Zhou X, Gao W, Huang H, Shao F, Liu Z (2020). A bioorthogonal system reveals antitumour immune function of pyroptosis. Nature.

[27] Ruan H, Leibowitz BJ, Zhang L, Yu J (2020). Immunogenic cell death in colon cancer prevention and therapy. Mol Carcinog.

[28] Keum N, Giovannucci E (2019). Global burden of colorectal cancer: emerging trends, risk factors and prevention strategies. Nat Rev Gastroenterol Hepatol.

[29] Center MM, Jemal A, Smith RA, Ward E (2009). Worldwide variations in colorectal cancer. CA Cancer J Clin.

[30] Ye J, Zhang R, Wu F, Zhai L, Wang K, Xiao M, Xie T, Sui X (2018). Non-apoptotic cell death in malignant tumor cells and natural compounds. Cancer Lett.

[31] Zhang Z, Zhang Y, Xia S, Kong Q, Li S, Liu X, Junqueira C, Meza-Sosa KF, Mok TMY, Ansara J (2020). Gasdermin E suppresses tumour growth by activating anti-tumour immunity. Nature.

[32] Schmitt AM, Chang HY (2016). Long noncoding RNAs in cancer pathways. Cancer Cell.

[33] Han P, Li JW, Zhang BM, Lv JC, Li YM, Gu XY, Yu ZW, Jia YH, Bai XF, Li L (2017). The lncRNA CRNDE promotes colorectal cancer cell proliferation and chemoresistance via miR-181a-5p-mediated regulation of Wnt/β-catenin signaling. Mol Cancer.

[34] Carpenter S, Fitzgerald KA. Cytokines and long noncoding RNAs. Cold Spring Harb Perspect Biol. 2018;10(6):a028589. 10.1101/cshperspect.a028589 PMC5983188 28716885

[35] Denaro N, Merlano MC, Lo Nigro C (2019). Long noncoding RNAs as regulators of cancer immunity. Mol Oncol.

[36] Tolba MF (2020). Revolutionizing the landscape of colorectal cancer treatment: the potential role of immune checkpoint inhibitors. Int J Cancer.

[37] Ooki A, Shinozaki E, Yamaguchi K (2021). Immunotherapy in colorectal cancer: current and future strategies. J Anus Rectum Colon.

[38] Boland PM, Ma WW (2017). Immunotherapy for colorectal cancer. Cancers (Basel).

[39] Li L, Jiang M, Qi L, Wu Y, Song D, Gan J, Li Y, Bai Y (2021). Pyroptosis, a new bridge to tumor immunity. Cancer Sci.

[40] Zhou Z, He H, Wang K, Shi X, Wang Y, Su Y, Wang Y, Li D, Liu W, Zhang Y (2020). Granzyme A from cytotoxic lymphocytes cleaves GSDMB to trigger pyroptosis in target cells. Science.

